# Simulation Study of Adhesive Material for Sandwich Panel under Edgewise Compression Condition

**DOI:** 10.3390/ma13061391

**Published:** 2020-03-19

**Authors:** Lanxin Jiang, Bing Yang, Shoune Xiao, Guangwu Yang, Tao Zhu, Dawei Dong

**Affiliations:** 1 State Key Laboratory of Traction Power, Southwest Jiaotong University, Chengdu 610031, China; jlanxin0530@163.com (L.J.); snxiao@swjtu.edu.cn (S.X.); gwyang@swjtu.edu.cn (G.Y.); zhutao034@swjtu.edu.cn (T.Z.); 2 Department of Thermal Power and Automotive Engineering, School of Mechanical Engineering, Southwest Jiaotong University, Chengdu 610031, China; dwdong@swjtu.edu.cn

**Keywords:** adhesive material, sandwich structure, finite element analysis, edgewise compression, tiebreak contact, MAT 185

## Abstract

In order to study the interfacial adhesive material simulation method of a sandwich structure with aluminum alloy panels and a low-density foam core under edgewise compression condition, two finite element models were defined using material model no. 185 (MAT 185) adhesive element and tiebreak contact, respectively, by LS-DYNA. Under the conditions of different loading rates, and element sizes, the effects of peak load, energy absorption, failure mode of adhesive layer and the influence degree of the changing condition on the calculated results were compared between the two models, and then compared with the experiment results and theoretical results. The higher the loading rate was, or the smaller the element size was, the higher the peak load was. The simulation results obtained using MAT 185 were closer to the experimental results under the edgewise compression condition.

## 1. Introduction

The traditional sandwich structure, which consists of two thin panels with high strength and low-density core, was firstly used in aircraft structures during World War II [1], is widely used in aerospace and rail transportation [2] at present. The sandwich structure consisting of aluminum alloy panel and foam core is extensively used in maglev train TR08 in Shanghai. Though sandwich structure has been used for decades, it is still necessary to study its properties under different load conditions with the development of technology. The property of interfacial material between panel and core is a main topic on research. Dogan et al. [3] compared the interlayer damage of fiber-reinforced composite material with MAT 185 and tiebreak contact under low-speed impact using LS-DYNA, and contrasted with experiment to get the optimal model. Meanwhile, it was proposed that the element size, number of material layers, and contact parameter have a great influence on the calculation results, and the follow-up research is necessary. Zheng et al. [4] simulated the interfacial adhesive between PMI foam and aluminum panel with cohesive element by Abaqus, which obtained good results. Lindström et al. [5] explored the failure mode of sandwich structure under the in-plane compression with boundary debonding, pointing out that the failure mode involves panel buckling, continuous debonding, and panel failure, which were complicated and related to the fracture toughness of the core material, the bending stiffness and strength of the panel, and the geometric characteristics. However, the influence of different adhesive layer simulation methods on the calculation results was not considered. Sleight and Lee et al. [6,7,8,9,10] studied the mechanical model of sandwich plates, in which adhesive layer cracked in the middle and end areas under in-plane load, and defined three typical failure modes. After adding stiffeners, the edgewise maximum load increased due to the less debonding and crack extension. Thomsen et al. [11,12,13,14,15] studied the buckling of sandwich plate with honeycomb core under edgewise load and the theoretical solution to predict buckling load. Honeycomb sandwich structure is also prone to interface debonding and panel buckling under edgewise load, so the adhesive simulation is equally important. Thus, the different core material also has an effect on the performance of the adhesive. Khan et al. [16] studied the in-plane and out-of-plane compression property of honeycomb core, whereas Marsavina [17] and Linul et al. [18] studied the bending performance, simulation method of Polyurethane (PUR) foam under different densities with different fracture criteria, and the energy absorption efficiency diagram under in-plane compression. All of them are instructive to research AIREX R82.80 Polyetherimide (PEI) foam used in this paper. Liu et al. [19,20] studied the finite element model of aluminum sandwich structure under high-speed bullet impact, and applied tiebreak contact to simulate the adhesive layer between panel and core. The results showed that the element size and bullet speed had a great influence on the calculation solution, while the response of other adhesive modeling method with different element size and loading speed (especially low speed) was not involved.

In summary, the existing studies on sandwich plate under edgewise compression mainly focused on carbon fiber panel and fiberglass panel with high-speed impact, and a series of results have been achieved. However, there consideration of the interface adhesive material between aluminum alloy panels and a low-density foam is still insufficient, and references on the optimal simulation method of adhesive at low speed are few. This paper mainly studied the sandwich plate consisting of 5754-H24 aluminum alloy panel and AIREX R82.80 low-density foam core under edgewise compression condition. Two kinds of modeling methods were utilized using cohesive element MAT 185 and tiebreak contact, respectively. The influences of the load rates and the element sizes on the calculated results were explored. Then compared with the experimental results, a better method was found for simulating the adhesive material.

## 2. Model and Material Theory

### 2.1. Finite Element Model

**Figure 1:**
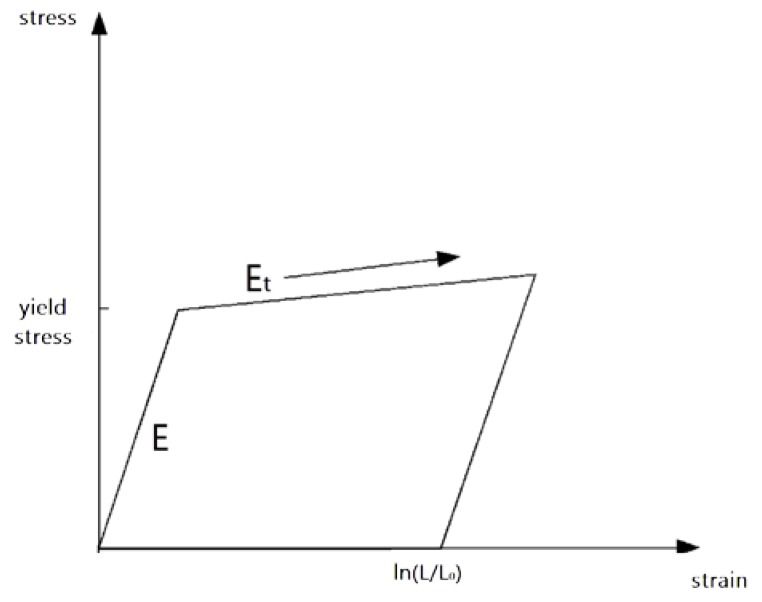
Elastoplastic bilinear material of MAT 3 [22].

When modeling sandwich plate, foam core was discretized by a solid element, and a low-density foam material MAT 57 [21] was adopted. The density, elastic modulus, stress–strain curve, and maximum tensile stress of the foam should be input for modeling. The characteristics of the foam were mainly determined by the stress–strain curve under compression by the test results. The panel was discretized by shell elements, and the elastoplastic bilinear materials of MAT 3 was adopted, as shown in Figure 1, where L and L_0_ were undeformed and deformed lengths of uniaxial tension specimen, respectively. E_t_ is the slope of the bilinear stress–strain curve. The elastic modulus, yield stress, and tangent modulus of the material were needed to be input.

For the sandwich structure under edgewise load, one of the key factors affecting the load-displacement curve, peak load, and energy absorption was the bonding state of the contact surface between the panel and core. In this paper, two kinds of cohesive elements were used. The first was MAT 185, which used the solid element only, and had good performance in simulating the behavior of adhesive [23]. The second was to set the tiebreak contact between the panel and core.

Due to the different manufacturing processes, raw materials, and other factors, the material parameters of aluminum alloy panels and foam core with the same brand were still different. In order to obtain accurate data of material parameters, the tensile test of the panel and compression tests of the core were carried out.

**Figure 2:**
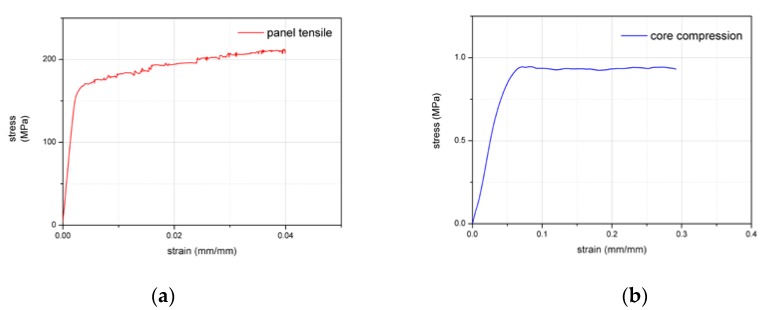
(**a**) Stress–strain curve of aluminum alloy panel and (**b**) stress–strain curve of foam core.

According to the ASTM C365-00 for the foam, Standard Test Method for Flatwise Compressive Properties of Sandwich Cores, and the ISO 6892-1-2009 for the aluminum alloy, Metallic Materials Tensile Testing Part 1: Method of Test at Room Temperature, using the MTS 647 Hydraulic Wedge Grip tester, with 0.5 mm/min displacement load, the stress–strain curves of 5754-H24 aluminum alloy panel and AIREX R82.80 foam core were obtained as shown in Figure 2a,b.

**Figure 3:**
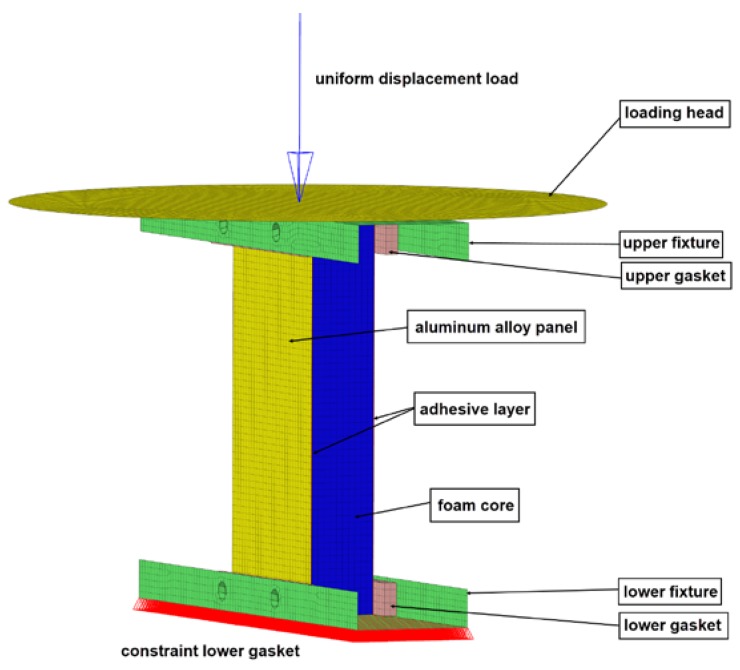
Finite element model of sandwich plate under edgewise compression load.

Input parameters were obtained from test curve; then the finite element model (Figure 3) was established whose fixture and loading head were referred to ASTM C364-99, Standard Test Method for Edgewise Compressive Strength of Sandwich Constructions. A uniform displacement load was applied at the loading head, and a fixed constraint was applied to the lower fixture. The loading head, fixture, gasket, and sandwich plate were set as surface-to-surface contact. The length of the whole structure was 120 mm, the width was 60 mm, and the thickness of the panel, the core, and the adhesive layer were 2, 20, and 0.1 mm, respectively.

### 2.2. Theory of Adhesive Layer Element

**Figure 4:**
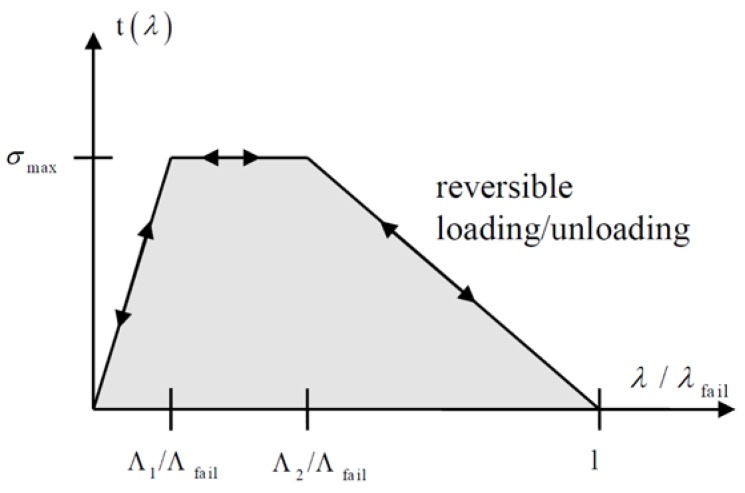
A trilinear constitutive relation of 185 material [22].

The first method to build adhesive material was using MAT 185, the behavior of which was defined by Tvergaard and Hutchinson [22,24]. It was a cohesive model only for solid element. In this material, a dimensionless separation parameter was defined to describe the interaction relationship between the relative displacement in normal and tangential directions as follows:

(1) $$\lambda =\sqrt{{\left(\frac{\delta_{1}}{TLS}\right)}^{2}+{\left(\frac{\delta_{2}}{TLS}\right)}^{2}+{\left(\frac{\delta_{3}}{NLS}\right)}^{2}}$$

where *NLS* is the maximum separation in the normal direction, *TLS* is the maximum separation in the tangential direction, $\delta_{1}$ and $\delta_{2}$ are the tangential relative displacements of mode II loading mode, and $\delta_{3}$ is the normal relative displacement of mode I loading mode. *NLS* and *TLS* are the key values, representing the maximum normal and tangential separation of the contact surface. The material is in a tensile state when $\delta_{3}\geq 0$, while in a compression state when $\delta_{3}\leq 0$. A trilinear constitutive relation according to λ can be defined as in Figure 4:

The constitutive relation is represented in the formula as:

(2) t(λ)={σmaxλΛ
1/Λfailλ<Λ1/ΛfailσmaxΛ1/Λfail<λ<Λ2/Λfailσmax1−λ1−Λ2/ΛfailΛ2/Λfail<λ<1

where $\Lambda_{1}$, $\Lambda_{2}$, and $\Lambda_{fail}$ are defined by users according to the material actual properties. When failure occurs (λ=1), the force acting on the element falls to 0, and the failed adhesive element is then deleted in the next load step.

For the second adhesive modeling method, the tensile failure stress, *NFLS*, and shear failure stress, *SFLS*, are required to be input as tiebreak contact set, and the failure criterion is [22]:

(3) $${\left(\frac{\left|\sigma_{n}\right|}{NFLS}\right)}^{2}+{\left(\frac{\left|\sigma_{s}\right|}{SFLS}\right)}^{2}\geq 1$$

If a more accuracy simulating state of the adhesive layer is needed, a curve about resisting stress versus gap opening for the post failure response should be input. When the adhesive judged to be invalid, the contact type is converted to normal surface-to-surface contact.

## 3. The Effect of Loading Rate and Element Size

Under the edgewise compression condition of sandwich structure, different loading rates have a great influence on the calculation results [19]. This section will explore the difference of load-displacement and energy absorption reflected by the two cohesive elements under different loading rates. 

### 3.1. The Effect of Loading Rate

**Figure 5:**
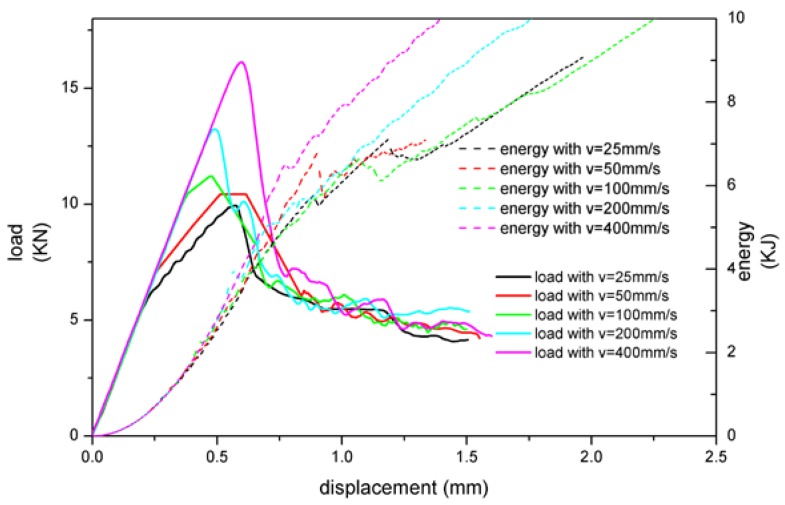
The relationship between load, energy absorption, and displacement with MAT 185.

**Figure 6:**
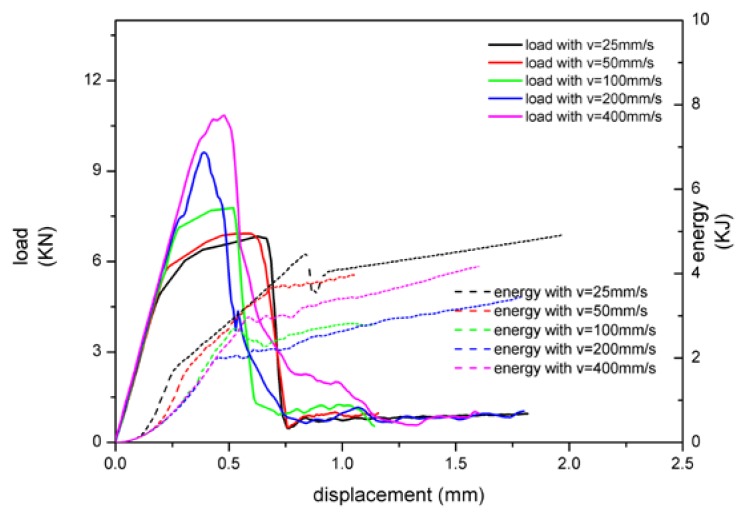
The relationship between load, energy absorption, and displacement with tiebreak contact.

The loading rate was set as 25, 50, 100, 200, and 400 mm/s, and the element size was 2 mm. The calculation results of the two modeling methods are shown in Figure 5 and Figure 6.

The overall trend of the load-displacement diagram was a linear increase in the early stage, and then, due to structural instability and buckling, the load decreased, then the adhesive layer was damaged, and finally, presented an approximate straight line. As can be seen from Figure 5, with the increase of loading rate, the peak load of the structure was also increased. At high velocity, the difference of calculation results was more obvious. At low velocity, it was easier for the structure to become unstable in the early stage when it was subjected to the edgewise compression, resulting in structural buckling which led to smaller peak load. From the perspective of energy, the model of MAT 185 absorbed less energy at low speed with similar value, while absorbed more energy with the velocity increase.

When the adhesive material was stimulated with tiebreak contact, the trend of load-displacement curve was basically the same as that of MAT 185. As the velocity increased, the peak load increased, and the peak value became more abrupt. When the velocity was small, the peak load remained unchanged for a period of displacement from buckling to adhesive failure as shown in Figure 6. In terms of energy absorption, the curve presented multilinearity, and the absorbed energy decreased first and then increased as the velocity was increased.

**Figure 7:**
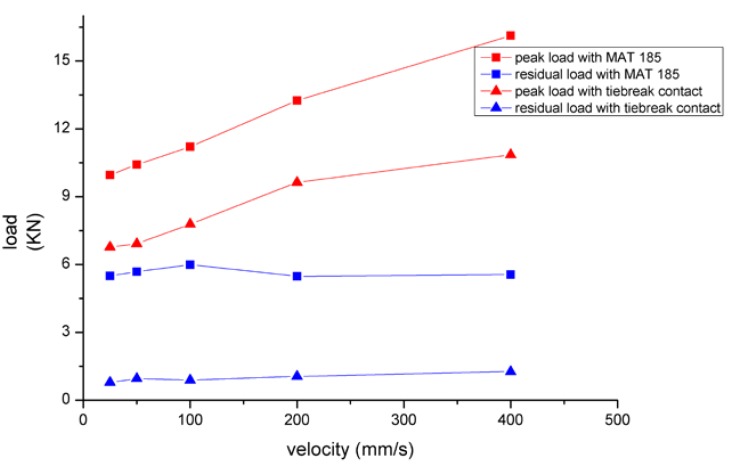
Comparison of peak load and residual load with MAT 185 and tiebreak contact.

As shown in Figure 7, the peak load values of the two modeling methods were obviously different. The peak load of MAT 185 was larger than tiebreak contact, and the residual load after the adhesive layer fails was also larger than tiebreak contact. For the changing of load rate, MAT 185 was more sensitive and the critical loads change more obvious. In terms of the energy absorption, the energy lines of MAT 185 tended to a straight curve, while that of tiebreak contact was multilinear. In addition, the amount of energy absorption varied with responses to the velocity. The main reason for these differences lied in the different sandwich plate failure modes presented by the two models.

**Figure 8:**
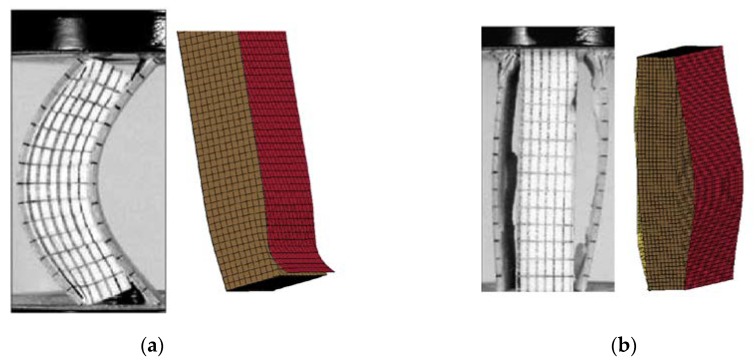
Failure mode with different finite element (FE) model. (**a**) Failure mode of mode I [8] and MAT 185 and (**b**) failure mode of mode II [8] and tiebreak contact.

In the relevant literature [8], scholars divided sandwich plate failure modes under edgewise compression into three types, namely, mode I, mode II, and mode III through a lot of tests. As shown in Figure 8a,b, the failure mode of MAT 185 was approximate to mode I, embodied in the boundary debonding of adhesive. The tiebreak contact was approximate to mode II, embodied the adhesive damage in the middle part, resulting in the debonding of entire panel. Due to the thorough damage of mode II, the structure was unable to support more load, so the energy absorption was also much smaller than mode I. The calculation results drew the corresponding conclusions.

### 3.2. The Effect of Element Size

According to the literature [3,13], element size has a great influence on the two FE models. In order to explore its change rule, element sizes were, respectively, set into 2, 3, and 4 mm, and the loading rate was 100 mm/s.

**Figure 9:**
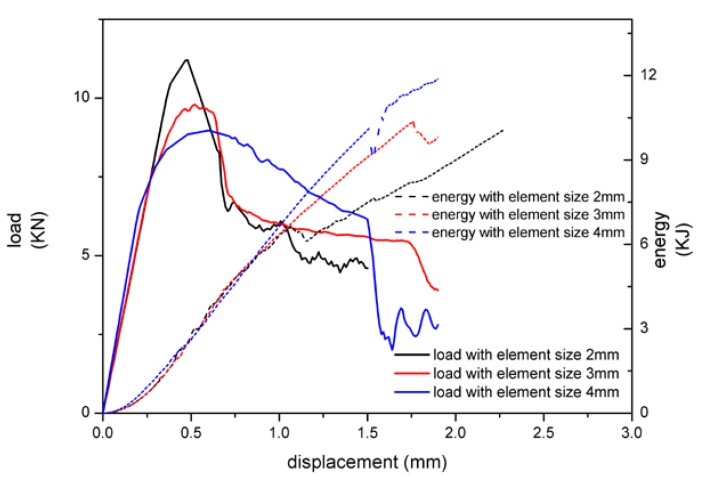
The relationship between load, energy absorption, and displacement with MAT 185.

With the increase of element size, the peak load decreased gradually, while the energy absorption increased with MAT 185 as shown in Figure 9. The structure was more prone to buckling when the element size was larger, so the peak load decreased. Meanwhile the failure of the adhesive layer was hard to occur with large element size, and the displacement was larger when adhesive failed.

**Figure 10:**
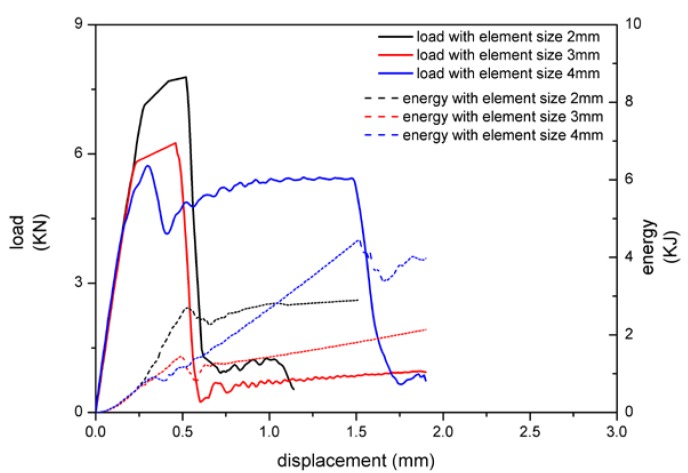
The relationship between load, energy absorption, and displacement with tiebreak contact.

Similar to MAT 185, the peak load decreased with the increase of element size, and the energy absorption decreased firstly and then increased with tiebreak contact as shown in Figure 10. Compared with the two results, the impact of element size of load-displacement curve and energy absorption was more obvious with tiebreak contact, which was reflected in larger peak load and energy fluctuation. The adhesive failure was not obvious when the element size was 6 mm, so the energy absorption increased sharply and the displacement during buckling stage was longer. The failure sensitivity of adhesive both decreased with the two modeling methods as large element size, but the tiebreak contact was more affected by it. Similarly, due to the different failure modes, the peak load and energy absorption of MAT 185 were higher than the tiebreak contact.

## 4. Simulation Results Compared with the Experimental Results and Theoretical Results

### 4.1. Experiment

According to ASTM C364-99, Standard Test Method for Edgewise Compressive Strength of Sandwich Constructions, the edgewise compression test of sandwich plate was carried out using MTS 647 Hydraulic Wedge Grip tester with 0.5 mm/min displacement load.

**Figure 11:**
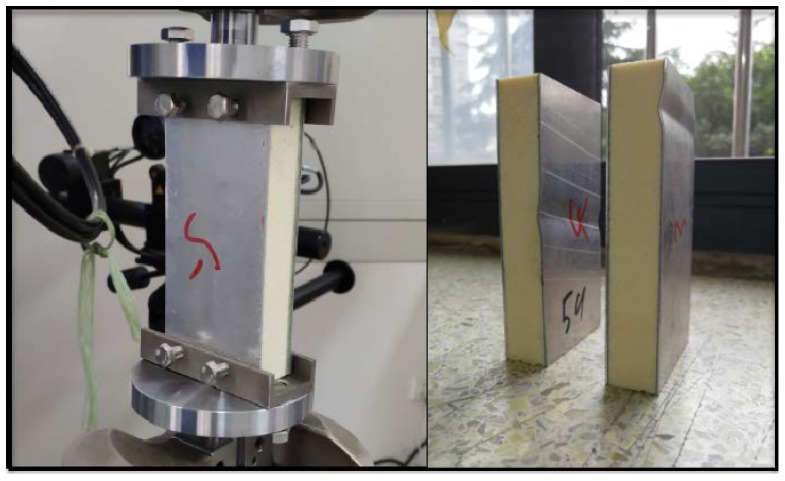
Edgewise compression test of sandwich plate and initial failure mode of buckling.

As shown in Figure 11, the width, length, and thickness of the sample are 60, 120, and 20 mm, respectively, which were consistent with the FE model. There were 5 samples in total. The two ends of the samples were constrained by the fixtures on the basis of standard, and the samples appeared buckling after compression, which was a typical initial failure mode of the structure.

**Figure 12:**
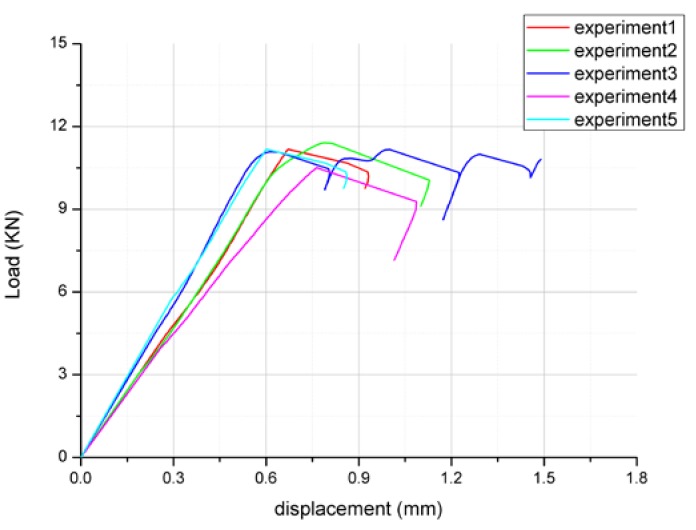
Load-displacement curve of sandwich plate.

The load-displacement curves of five samples are given as Figure 12. Due to manufacturing differences, the five curves were not the same, but the overall results were consistent. The test results were similar to the calculation results, showing that the load increased linearly on early stage, then reached the peak load and buckling. Finally, the load decreased with the increase of displacement.

### 4.2. Comparison of Results

According to the theoretical solution method of sandwich edgewise compression peak load, the structure appears unstable buckling when load reaches the Euler load, and Euler load $P_{E}$ could be calculated by the following formula:

(4) $$P_{E}=\frac{\pi^{2}D}{{\left(\beta L\right)}^{2}}$$

where *D* is bending stiffness and *L* is the length of the structure. β is a boundary coefficient, and the value depends on whether the boundary is supported or not. In this test, there were clamps on both sides of the boundary, so 0.5 was taken for β.

The edgewise load would cause shear deformation of the foam core, resulting the peak load being less than the Euler load. Ignoring the local bending stiffness of the panel, the bending stiffness of the structure is

(5) $$D=E_{f}btd^{2}/2$$

where $E_{f}$ is the Young’s modulus of the panel, *b* is the width, *d* is the total thickness of the structure, *t* is panel thickness, and the peak load is expressed as:

(6) $$P=\frac{P_{E}}{1+\left(P_{E}/AG\right)}$$

where $A=\frac{bd^{2}}{c}$, *c* is the thickness of core material, and *G* is the shear modulus of core material. When *G* is infinite, the peak load is close to Euler load, and when *G* is small, the peak load is close to *AG*, which is, the shear stiffness of core material.

Without considering the influence of adhesive layer on the modulus of compression direction, the theoretical modulus of compression direction is

(7) $$E=E_{f}V_{f}+E_{c}V_{c}$$

where $E_{c}$ is Young’s modulus of core material, $V_{f}$ is the volume fraction of the panel, and $V_{c}$ is the volume fraction of the core.

**Figure 13:**
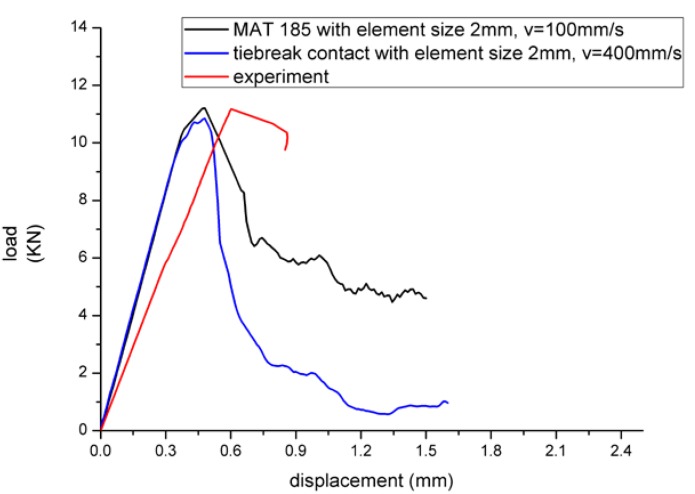
Comparison of simulation result and experiment result.

The modulus of MAT 185 on compression direction was closer to the experimental value, and the difference between the two models was not significant as shown in Table 1. The theoretical modulus did not considered the influence of adhesive layer, so the value was relatively large. From the perspective of peak load shown in Table 2, tiebreak contact was generally small for the structure which was more unstable and prone to buckling. In addition, the theoretical solution of peak load was relatively large without considering the adhesive layer. In general, the peak load was closer to the test when the velocity was 100 mm/s and element size was 2 mm with MAT 185, and the velocity was 400 mm/s and element size was 2 mm with tiebreak contact. Therefore, the two groups of data were selected to be compared with the load-displacement curve of the third sample, as shown in Figure 13. From the point of peak load, MAT 185 was closer to the test value. On the other hand, the test was carried out at low velocity, so the MAT 185 with v = 100 mm/s was more realistic. In the elastic stage, both of the two models had discrepancy as a result of the measurement error on the adhesive thickness, which had a great influence on the structure. At the same time, the parameters of the adhesive material were inaccurate.

**Figure 14:**
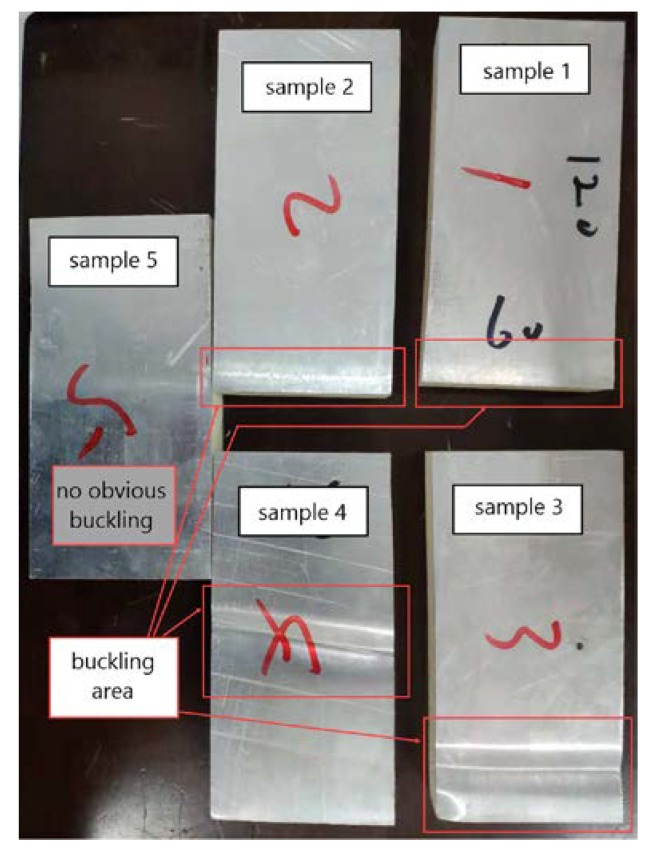
The deformation positions of 5 samples after compression.

From the perspective of geometric deformation, the results of the five samples were not the same. Sample 1 and sample 2 buckled at the end position, sample 3 buckled at the top position and the end position, sample 4 buckled at the middle position, and 5 did not buckle significantly after the load dropped as shown in Figure 14. Compared with the out-of-plane compression of sandwich structure, there were many factors that influenced the edgewise compression, such as manufacturing difference, defect of adhesive layer, and bolt tightening torque of test fixture. All these factors resulted in the difference of sample deformation.

**Figure 15:**
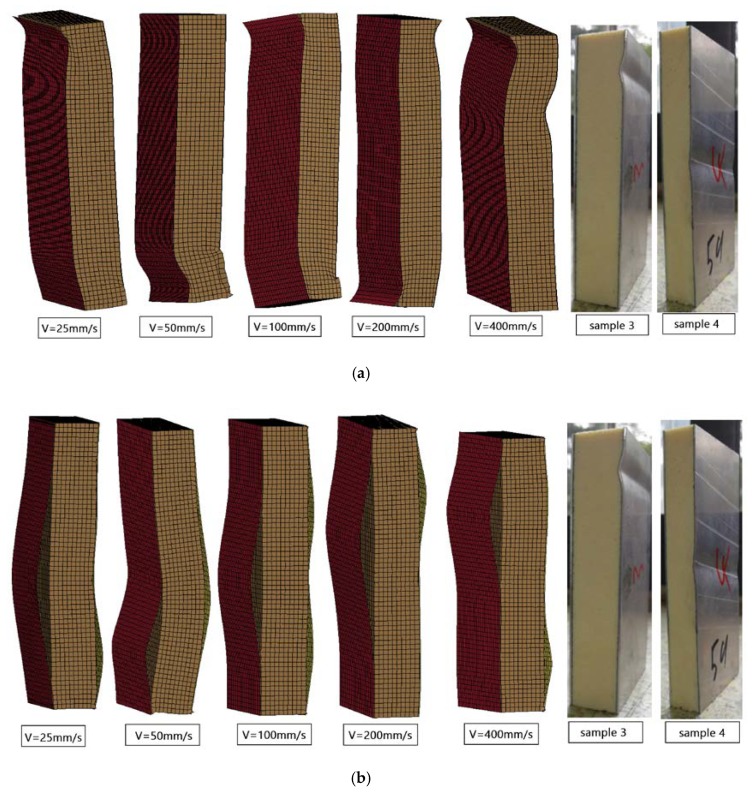
(**a**) Simulation and experimental deformation diagrams at different loading speeds with MAT 185 and (**b**) simulation and experimental deformation diagrams at different loading speeds with tiebreak contact.

The same deformation difference was also reflected in the simulation results. As shown in Figure 15a,b, the deformation positions of the structure were different at the same time when the loading speed is different. MAT 185 buckled at the end on low speed and the buckling area moved to the middle at a rapid loading speed. The failure mode of tiebreak contact was the large area debonding between panel and core, and the initial positions of debonding with different speeds were also different without obvious rules. Compared with the test results, sample 3 and sample 4, the failure mode of MAT 185 was more similar, which was embodied as the end and middle buckling.

On the whole, the result was more similar to the test result when using MAT 185 to simulate the edgewise compression of sandwich structure consisting of foam core and aluminum alloy panel. The model of tiebreak contact was prone to buckling with the larger error, while the advantage was the smaller calculation amount, which could save computing time and memory.

## 5. Conclusions

In order to study the simulation method of adhesive material between aluminum alloy panels and a low-density foam core under edgewise compression condition, namely, load-displacement relationship and energy absorption, two kinds of cohesive elements were applied using MAT 185 and tiebreak contact, respectively, for finite element simulation. Different loading velocities and element sizes were set to compare with the calculated results. Then, the experiment was conducted to verify the calculated results. The following conclusions could be drawn:

Under different loading rates, peak load, residual load, and energy absorption of MAT 185 were all higher than the tiebreak contact. This was because the model of tiebreak contact was more prone to structural buckling, and the two models presented two different failure modes, boundary debonding and panel debonding. The peak load of the two models increased with the increase of the loading rate, while MAT 185 was more sensitive to the rate change.

As for different element size, the peak load of the two modeling methods decreased with the increase of element size. At the same time, the sensitivity of adhesive failure decreased when the element size increased with the adhesive damage appeared even later, leading to the energy absorption increase. The change of element size had a greater impact on the model of tiebreak contact.

Compared with the test data, there was a certain error due to the thickness and the accuracy material parameters of the adhesive layer. In the case of small element size, MAT 185 was closer to the actual situation, while tiebreak contact needed higher loading rate to be fitted with the test results. In terms of deformation, MAT 185 was more consistent to the test as buckling in the end and middle area. Therefore, the interfacial adhesive layer was more suitable for simulation with MAT 185 when the sandwich structure was composed of aluminum alloy panel and a low-density foam core under edgewise compression.

Due to the limitation of test conditions, the energy absorption by the structure has not been recorded, and the failure position of the adhesive layer after buckling and the final failure mode have not been discussed yet. In addition, the influence of foam materials, thickness of panel and interfacial adhesive layer on load-displacement characteristics, energy absorption, and failure mode still need to be further studied.

## Figures and Tables

**Table 1:** Comparison of simulation, experiment, and theoretical results with modulus.

Material Parameter	MAT 185	Tiebreak Contact	Test	Theory
Modulus of compression direction/MPa	2569	2579	1698	3544

**Table 2:** Comparison of simulation, experiment, and theoretical results with peak load.

Material Parameter			MAT 185	Tiebreak Contact	Test	Theory
Peak load	Velocity	25 mm/s	9.95 KN	6.77 KN	11.05 KN	18.32 KN
		50 mm/s	10.42 KN	6.92 KN		
		100 mm/s	11.21 KN	7.78 KN		
		200 mm/s	13.25 KN	9.63 KN		
		400 mm/s	16.13 KN	10.85 KN		
	Element size	2 mm	11.2 KN	7.78 KN		
		3 mm	9.79 KN	6.26 KN		
		4 mm	8.97 KN	5.73 KN		
